# Improving the Breadth of the Host’s Immune Response to Lassa Virus

**DOI:** 10.3390/pathogens7040084

**Published:** 2018-10-28

**Authors:** Juan Carlos Zapata, Sandra Medina-Moreno, Camila Guzmán-Cardozo, Maria S. Salvato

**Affiliations:** Institute of Human Virology, School of Medicine, University of Maryland, Baltimore, MD 21201, USA; smmoreno@ihv.umaryland.edu (S.M.-M.); mcguzmanc13@gmail.com (C.G.-C.); MSalvato@ihv.umaryland.edu (M.S.S.)

**Keywords:** Lassa virus disease (LVD), vaccine breadth, mimicry, B cell anergy, conserved antigens, Fc-gamma receptors, conformational antigens, broadly-neutralizing antibodies, focused immunity, dominant and subdominant epitopes, cross-restriction

## Abstract

In 2017, the global Coalition for Epidemic Preparedness (CEPI) declared Lassa virus disease to be one of the world’s foremost biothreats. In January 2018, World Health Organization experts met to address the Lassa biothreat. It was commonly recognized that the diversity of Lassa virus (LASV) isolated from West African patient samples was far greater than that of the Ebola isolates from the West African epidemic of 2013–2016. Thus, vaccines produced against Lassa virus disease face the added challenge that they must be broadly-protective against a wide variety of LASV. In this review, we discuss what is known about the immune response to Lassa infection. We also discuss the approaches used to make broadly-protective influenza vaccines and how they could be applied to developing broad vaccine coverage against LASV disease. Recent advances in AIDS research are also potentially applicable to the design of broadly-protective medical countermeasures against LASV disease.

## 1. Introduction

Lassa virus (LASV) is a zoonotic pathogen endemic to West Africa. Annual outbreaks occur primarily during the dry season amongst the rural population [1]. In December 2013, West Africa experienced an outbreak of Ebola virus that quickly grew into an epidemic, revealing that much of this part of the world was unprepared to handle such a disaster. The Ebola epidemic was a more terrifying version of the annual Lassa disease outbreaks, and was characterized by high person-to-person transmission, the deaths of medical personnel, popular hysteria and occasional transmission outside West Africa [2]. It became clear that the unchecked spread of such infections could endanger the rest of the world. 

In 2017, the global Coalition for Epidemic Preparedness (CEPI) declared Lassa virus disease to be one of the world’s foremost biothreats [3]. In January 2018, the World Health Organization (WHO) convened a “Lassa Roadmap” panel lead by Mike Osterholm, an epidemiologist and expert in biosecurity [4]. As the panel considered the technical obstacles to Lassa vaccine production, one of the more important obstacles was “breadth of vaccine protection”. Breadth refers to the variety of infections suppressed by one vaccine. If the vaccine is broadly protective, it might shield vaccinees from all of the genetically diverse Lassa lineages identified in patient samples [5]. Sequencing studies of blood from infected West African patients showed an almost 50-fold greater variation of LASV isolates than of Ebola virus isolates [6,7]. This means that a Lassa vaccine needs to be more broadly protective than an Ebola vaccine because it needs to cover a greater genetic variation. This variation is presumably due to the fact that most of the Lassa patients are infected directly through the rodent reservoir, whereas most Ebola patients are infected by contact with other human patients. Hence, the high variation of Lassa outbreaks is due to multiple introductions from the rodents and not to any changes in viral mutation rate. Another contributing factor may be that LASV replication in culture generates 10–1000-fold fewer virus particles per infectious particle than Ebola virus Zaire or Sudan isolates [8]. This means Ebola virus produces an excess of viral products and only a few of them assemble to become infectious particles. 

Vaccine cross-protection reflects the genetic diversity covered by a vaccine. If a Lassa vaccine is “narrowly protective”, it will only protect against one or two Lassa lineages: two closely-related lineages differ in 5–7% of their nucleotide sequence. If the vaccine is more broadly protective, it could protect against all Lassa lineages (the most divergent LASV lineages differ by as much as 27% of their nucleotides) [9]. If the vaccine is even broader, it should protect against other arenavirus species. The genetic diversity between LASV species and a closely related Old World arenavirus species such as LCMV ranges 30–50% difference between their nucleotide sequences [9]. 

The last 50 years of LASV research shed some light on its pathogenesis and effects on the host immune response. During the early innate response, LASV infection seems to affect dendritic cell function, resulting in poor antigen-presentation, partial immunosuppression and unchecked virus replication [10,11,12]. In monkeys and in Lassa-infected people, neutralizing antibodies are slow to develop, partially due to glycans on the viral envelope (GP) that mimic self-glycans and anergize the B cell response [13].

Several LASV vaccine candidates have demonstrated efficacy in animal models. A live attenuated MOP/LAS reassortant vaccine (clone ML29), designed by Lukashevich and further developed to pre-clinical studies in our laboratory, has shown sterilizing protection against Lassa disease in mice, guinea pigs, and non-human primates [14,15,16,17,18]. Additionally, this vaccine elicits immune responses to LASV glycoprotein (GP) and nucleoprotein (NP), even in primates chronically-infected with SIV [19]. In this review, we discuss the Lassa vaccine candidates that have demonstrated broad cross-protection and the key properties contributing to their breadth. 

The goal of this review is to discuss possible ways to improve the immune response to LASV. In the first part, we discuss what is known about the development of immune responses to Lassa virus and the current vaccine candidates used to confer antiviral immunity, then we describe the approaches used to obtain “universal flu vaccines” and broadly protective AIDS vaccines, and how these approaches could possibly be applied to medical countermeasures against Lassa fever.

## 2. Clinical Manifestations and Pathogenesis of Lassa Virus Disease

**Figure 1:**
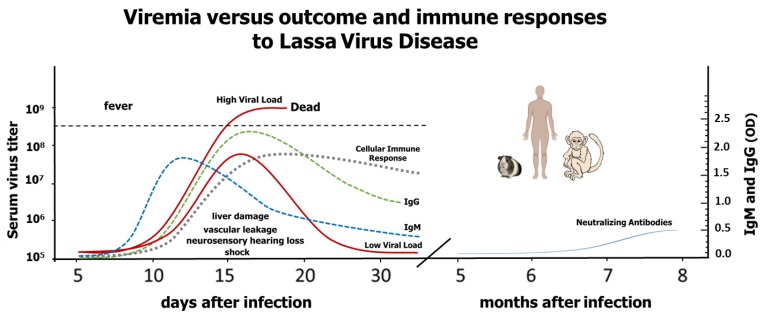
This is a representation of LASV viremia in relation to Lassa virus disease outcomes and immune responses to Lassa virus based on published data about rodent, non-human primate and human infections. When the immune system fails to control the virus, disease is more acute and leads to death. Those individuals with moderate viral replication (~80%) are either asymptomatic or, if they develop symptoms, they have higher possibilities to survive (low solid red line), while those patients with high viral loads suffer severe disease that can lead to death (high solid red line) [21,23]. Dotted lines represent cellular and humoral immune responses. Solid blue line represents the rise of neutralizing antibodies.

LASV pathogenesis and its failure to develop strong immune responses remain a mystery. Although there is a strong correlation between the level of viremia and the disease outcome (Figure 1), the damage is not caused directly by viral-cell lysis and seems to depend on the initial host immune response [20,21,22]. The first symptoms are poorly differentiated from other diseases. In addition, the incubation period can be as long as two weeks. Those two situations make the initial diagnosis difficult and cause a delay in the initiation of the treatment. Twenty percent of the infected patients develop symptoms of muscle fatigue, facial edema, and sore throat, and a few of these progress to systemic disease with mucosal, conjunctival, gastrointestinal or genital bleeding. Platelet dysfunction and endothelial damage seem to play a role in the characteristic vascular leakage.

Fatal cases are associated with myocarditis, pulmonary edema, acute respiratory distress, and a hypovolemic shock; in addition, elevated plasma aminotransferases (AST and ALT), uncontrolled viremia, and high levels of IL-6 are pathognomonic for LF [22]. Massive viral replication in the liver and spleen leads to progressive hemorrhagic manifestations and increased mortality. It is common to find hepatocellular necrosis and foci of hepatocyte proliferation [24,25,26]. A recent description of arenavirus-induced liver pathology was characterized by hepatocytes with increased cell death, upregulated cell cycling factor p21, IFN-γ, and LASV receptor Axl-1, but aborted cell cycling. Whereas mature hepatocytes had low alpha-dystroglycan (α-DG; a LSV receptor) expression, oval cells had high expression of α-DG [27]. Coagulation disorders are not common in LF; however, when they happen, platelet counts could be normal with little disseminated intravascular coagulation, while platelet aggregation is impaired and loss of liquids [28,29,30,31,32]. Neuropathological manifestations include disorientation, motor and sensory abnormalities, convulsions, hiccups, and in advanced stages coma. Brain dysfunctions are associated with poor prognosis and it is not clear if they are the result of the direct effect of LASV, nonspecific metabolites, or immune-mediated effects. LASV infection is responsible for the high prevalence of hearing loss in West Africa: around 30% of LF patients develop hearing problems and 17% of LF survivors suffer permanent hearing loss [33,34,35,36,37]. 

## 3. Early Immune Response to Lassa Virus Infection 

LF survivors are able to control viral loads early during the infection. In contrast, fatal cases show poor inflammatory responses characterized by lymphopenia, that affects all lymphocyte subpopulations, including CD4+ and CD8+ T cells, B cells, and NK cells, along with necrosis of lymphoid organs [29,38,39,40,41]. This immuno-suppressive state appears to be induced early during the infection. In vitro LASV infection of macrophages, dendritic cells (DC), and endothelial cells down-regulated the production of inflammatory mediators [10,11,42]. In vivo, antigen presenting cells (APC; macrophages (MP) and dendritic cells (DC)) are the primary targets of LASV. In monkeys, by seven days after infection, infected DC were found in a variety of tissues. MP were also infected to a lesser extent. Kupffer cells, hepatocytes, adreno-cortical cells and endothelial cells were more frequently infected in the tissues of terminal animals. In lymph nodes, LASV antigen was detected in DC located in the marginal zone and to a lesser extent in monocytes and MP in the marginal zone and red pulp [40].

Most RNA viruses activate DC and MP while replicating due to the production of dsRNA genomic intermediates and other viral sub-products presenting pathogen molecular patterns (PAMPs), which are recognized by specialized pattern recognition receptors (PRR). LASV PAMPs activate several signaling cascades that lead to the secretion of chemokines and cytokines, including interferon (IFN) responses that inhibit virus replication and induce cellular genes involved in innate and adaptive immunity [43,44]. Although sensitive to the antiviral effects of IFN-α and IFN-γ, LASV has been shown to regulate IFN production in vitro and in vivo [10,11,42,45]. In lethally-infected animals, IFN-γ levels were moderately elevated [46] and, in human fatal cases, IFN-γ levels were increased in some individuals [47]. In macaques, IFN type I was detected very early in survivors but only in the late stages of fatal cases [38]. The inhibition of IFN by LASV affects plasmacytoid DC maturation and reduces production of cytokines and chemokines by these cells [43,48]. Another function inhibited by the block to DC maturation is their capacity to migrate to the secondary lymphoid organs, to express CCR7, and thus to activate T cells [11]. 

A common feature of the viral nucleoprotein (NP) of mammalian arenaviruses is its ability to prevent nuclear translocation of interferon regulatory factor 3 (IRF-3), IFN type I activation, and the downstream induction of the interferon stimulated genes (ISG) [48,49,50,51]. The anti-IFN activity of NP is located in its C-terminus that is structurally homologous to the DEDDH family of 3′-5′ exoribonucleases [52]. The NP crystal structure and its interaction with RIG-I and MDA-5 suggest that NP ribonuclease activity is able to remove viral PAMP RNA, thereby avoiding recognition by PRRs and inhibiting IFN production [52,53]. The LASV Z matrix protein also binds RIG-I and MDA-5 to inhibit type I IFN induction [54]. In contrast to NP, Z proteins of some arenaviruses do not bind RIG-I/MDA-5 and fail to inhibit IFN production [54]. The authors suggest that Z-mediated anti-IFN activity is more frequently associated with pathogenic arenaviruses. Another group, showing that the L polymerase is subject to more positive selection events (>dN/dS) during evolution than any other arenaviral gene, argues that the polymerase might be central to viral pathogenesis [55]. 

LASV infection of monocyte and endothelial cell cultures suppressed TNF-α and IL-8, an effect that in vivo would be predicted to inhibit inflammation and neutrophil migration [10]. In contrast, infected monocyte-derived DC showed an increase in IL-8 secretion with a reduction in the expression of co-stimulatory molecules such as CD86, CD80, and CD40. Additionally, LASV-infected DC failed to produce pro-inflammatory cytokines and to stimulate T cells [42]. Although both MP and DC are susceptible to LASV infection, immature DC supported virus replication without being destroyed or activated. Neither the infection of human DC and MP nor their stimulation with inactivated virus induced the production of TNF-α, IL-1β, IL-2, IL-6, IL-8, IL-10, IL-12p35, TGF- β, IFN-γ, or CD25 [11,42]. 

In line with the cell culture results, LF fatal cases see a reduction in IL-8 and IP-10 in comparison to survivors [11,42]. Similar results were seen in vivo in cynomolgus macaques [40]. IL-8 was the only cytokine that peaked in infected people who did not develop LASV disease [56]. In addition, primates and human PBMC exposed to LCMV-WE (a virus causing hemorrhagic fever) showed that virulent infection was associated with undetectable levels of TNF-α, low levels of IL-8 in plasma and inhibition of IL-8 mRNA expression [46,57]. 

In vitro, LASV and LCMV-WE down-regulated IL-6 and other pro-inflammatory cyto/chemokines in human MP and epithelial cells. In contrast, MOPV and LCMV-ARM strongly up-regulated pro-inflammatory responses in a TLR-2/Mal-dependent manner [58]. Meanwhile, a high level of IL-6 in plasma is a biomarker of progressing LF in humans and in primates experimentally infected with LASV or LCMV-WE. This high level of IL-6 during the last stages of severe LF could result from hepatic regeneration and may be associated with neutrophilia [22,24,38,46,59]. We speculate that the in vitro studies model the earliest events in LASV infection, whereas the IL-6 in infected macaque plasma is a late event of LASV infection; hence, severe pathology would be marked by early suppression of IL-6 and late abundance of IL-6. 

Taken together, the above evidence suggests that LASV targets monocytes and DC, inhibits the initial immune response and suppresses the migration of activated cells to the primary infection site resulting in higher viral replication, greater virulence, and a delay in the induction of the acquired immune response.

## 4. Acquired Immunity to Lassa Virus Infection

LASV-specific IgM and IgG are detected during peak viremia, and appear unrelated to recovery from disease [20]. Primate immunization with gamma-irradiated LASV, an inactivation procedure that preserves the natural structure of antigens, induced strong antibody responses to NP and GP antigens, but failed to protect immunized animals from fatal LF [60]. Notably, NP is the earliest antigen detected by antigen-capture assays in infected individuals, most likely because it is the most abundant structural component of each virion [61]. In addition, ELISA to detect NP antibody was used in early seroprevalence studies [62]. Due to the structural importance of NP, it is likely to contribute to the breadth of the acquired immune response to LASV (see arguments in Section 6). 

In cynomolgus macaques experimentally infected with LASV, the antibody titers against GP1, GP2, and NP increased more rapidly in survivors than in fatal cases [38]. Neutralizing antibodies (nAbs) in convalescing people can be detected only in low titers, several months after the initial infection and have been LASV strain-specific [23,63]. These findings suggest that the antibody response may not be responsible for the patient’s recovery. However, antibodies could be playing an important role in attenuating acute infection, as suggested by its early appearance in surviving monkeys, the high seroprevalence in endemic areas without clinically overt disease, the lack of disease in infected contacts or the high mortality rate in individuals from LASV-free regions [38,56,64].

Treatment of human beings and animals with immune-antiserum showed a range of results [23,65,66,67]. A study testing a cocktail of human nAbs, generated in vitro from LASV_Josiah_-infected individuals, was able to rescue late-stage infected macaques from death. In those experiments, viremic macaques were treated with a single dose of the cocktail at Days 6 and 8 after LASV infection leading to virus clearance and survival [68]. Although antibody cocktails of 15 mg nAb /kg macaque constitute an expensive treatment, their success is an important proof-of-concept. Recent structural studies revealed that artificially-generated nAbs recognized a metastable pre-fusion GP complex and blocked changes required for engagement with the intracellular receptor LAMP-1 and fusion with host membrane in late endosomes [69]. However, LASV infection induces predominantly non-nAbs against conserved NP and GP2 antigens and with a few exceptions these antibodies become undetectable after several months [62,70]. Seronegative survivors (approximately 18% of total within the LASV endemic areas) were not resistant to LASV re-infection but were protected from clinical LF [71]. 

After acute LF recovery, patients overcome the initial lymphopenia and develop a strong CD4+ immune response against NP and GP2. The NP-CD4+ response is only partially strain-specific since it cross-reacts with other LASV strains [72]. The LASV-GP2-CD4+-specific response recognizes a conserved epitope that is common in the Old World (100% similarity with LCMV) and New World arenaviruses (>90% similarity) [73]. In animal models, T cell responses are more important than B cells responses, and the CTL-mediated protection seems to rely more on CD8+ cells than on CD4+ cells [19]. In LASV-infected monkeys T-cell activation was delayed in fatal cases and in vitro stimulation of lymphocytes from those animals did not result in proliferation [38]. There were also decreases in CD20+ cells, and down-regulation of class II MHC antigens [25]. In contrast to fatal cases, survivors showed activation and proliferation after exposure to inactivated LASV, as well as an increase in circulating monocytes [38]. Similarly, ML29 immunization of marmosets increased the number of CD3+ and CD14+ cells [25]. 

The LASV-infection of one health care worker gave the USA Centers for Disease Control the rare opportunity to monitor blood cells during disease progression in a human patient [74]. During the acute phase, CD4 and CD8 T cells peaked in conjunction with virus clearance. During convalescence, the CD4 T cells waned while CD8 T cell activation and degranulation peaked for a second time. The patient was ultimately able to generate long-term, polyfunctional, Lassa virus-specific T cells, with approximately 66% of the CD4 T cells and 75% of the CD8 T cells expressing more than one cytokine. Taken together, these results suggest strong participation of T cell responses in protection and recovery during natural LASV infection. However, despite recent successful recoveries, the mechanism of protection remains unknown and more studies of human cases are needed to characterize the role of T cells in LF protection [15,41,70,75,76,77]. The evidence suggests that the inhibition of pattern recognition receptors (PRR) by LASV at the beginning of the infection, and the failure to induce early pro-inflammatory cytokines results in a temporary immunosuppression and uncontrolled virus replication. An early and strong immune response that controls virus replication is more likely to promote recovery from LASV disease.

## 5. A comparison of Promising Lassa Vaccine Candidates

**Figure 2:**
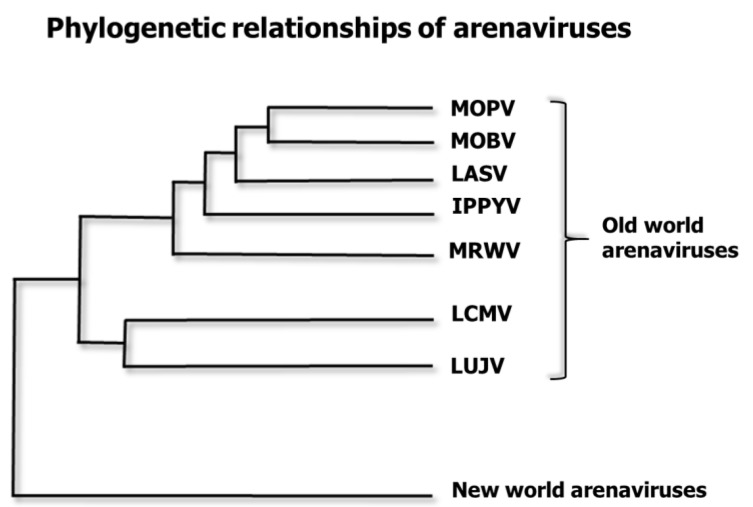
Phylogenetic relationships of some Old-World arenavirus species. LASV is related to several “Old World” arenaviruses. Mopeia virus AN20410 (NC_006574.1) is designated MOPV. Mobala virus (NC_007904.1) is MOBV. Lassa virus (NC_004297.1) is LASV. Ippy virus (NC_007906 is IPPYV. Merino Walk Virus (NC_023763.1) is MRWV. Lymphocytic choriomeningitis virus (NC_004291.1 is LCMV. Lujo virus (NC_012777.1) is LUJV. The family *Arenaviridae* has three genera: *Mammarenaviruses*, *Reptarenaviruses* and *Hartmaniviruses* [80]. Here, we only depict seven species of the Old-World group of the *Mammarenavirus* genus, omitting the New World *Mammarenaviruses* and the other two genera. This is a maximum clade credibility tree of the polymerase region. The tree was constructed from amino acid alignment using Bayesian MCMC method with LG model of substitution.

Immunization of monkeys and guinea pigs with LCMV, MOBV, MOPV, and other non-pathogenic Old-World viruses can all confer some protection against LF disease [15,19,41,70,75,76,78] (Figure 2). Mopeia virus (MOPV), an arenavirus species found in rodents of eastern and southern Africa, serves as a naturally attenuated vaccine, protecting non-human primates from a lethal challenge with LASV [78,79]. The fact that MOPV and LASV can cross-protect, even though they belong to different species shows that broadly-protective vaccines are feasible. Knowledge that MOPV is a widespread, cross-protecting and non-pathogenic virus led to the production of at least two vaccines based on the MOPV platform (ML29 and MOP-VAC).

In this section, we compare five Lassa vaccine candidates with respect to their estimated economy of production, safety for pregnant women, breadth of protection, and capacity to confer sterilizing immunity after a lethal challenge (Table 1). Published claims and extrapolated guesses have been used for these estimates, since no pair of these vaccines has yet been put to a rigorous head-to-head comparison. Such a comparison should eventually be done for the benefit of all stakeholders.

ML29, derived from lineage IV Lassa_Josiah_, is considered a broadly cross-protective Lassa vaccine because it showed sterilizing immunity in guinea pigs challenged with a distantly-related lineage II strain of Lassa virus [5,16]. Broad cross-protection by ML29 was also observed in SIV-infected rhesus macaques given a lethal challenge with LCMV-WE. Whereas naive macaques given LCMV-WE succumbed to a LF-like febrile illness, four macaques immunized with ML29 survived for a month without increases in plasma AST or ALT (MSS unpublished, with remaining animals from [19]). This finding was consistent with the previous observation that ML29-inoculated rhesus macaques induced strong cross-reactive cell-mediated immunity to LCMV-WE [15]. 

The VSV-Las vaccine is a rhabdovirus vector expressing a single Lassa gene, the GPC. Guinea pigs vaccinated with VSV-Las_Josiah_ were challenged with two closely-related strains, Liberian Lassa_Z-132_, and a Malian Lassa_SorombaR_. Currently, there is no challenge model for LASV from lineage I LASV_Pinneo_, so in these experiments all animals survived including the challenge controls, making it impossible to justify claims about cross-protection from LASV_Pinneo_. Three cynomolgus macaques vaccinated with VSV-Las_GP_ and challenged with the related lineage IV strain, Lassa_Z-132_, also survived. Monkey challenges with distantly-related strains such as LASV_Pinneo_ were not reported. Thus, in contrast to ML29, the VSV-Las_GP_ has demonstrated only narrow protection against LASV lineages closely-related to the vaccine and has failed to demonstrate sterilizing immunity [81].

Lassa vaccine testing in guinea pigs has given misleading positive results in the past, for example a LasNP vaccine was able to protect guinea pigs but not primates [82]. Cross-protection between LCMV and LASV in guinea pigs was less effective in primates [83]. Vaccines constructed on the yellow fever vaccine platform, YF17D, were found to work well in guinea pigs but were relatively disappointing in primates. It was discovered that the YF-Las_GP1_ and YF-LAS_GP2_ vaccines were more stable than the YF-Las_GPC_ vaccine and protected 80% of guinea pigs [84,85] but failed to protect marmosets (I. Lukashevich, unpublished). We would speculate that the best cross-protection can be achieved only with the most stable and conserved particle structures. Additional Lassa antigens, such as NP or Z, which provide conserved epitopes as well as increase the formation of stable ribonucleoprotein particles, are predicted to increase vaccine breadth. A rigorous test of vaccine cross-protection or breadth must ultimately occur in primates.

The laboratory of Silvain Baize has recently described a new vaccine (MOPVAC_LasGP_) that is a Mopeia recombinant bearing a Lassa_Josiah_ glycoprotein (GP). It has been genetically engineered to have six missense mutations in the exonuclease portion of its nucleocapsid protein (NP), a portion that is known to suppress the antiviral IFN response by cleavage of PRR-detecting RNA. This MOPVAC has not been tested for cross-protection, but it is seen as a platform for insertion of sequences for each of the Lassa lineages. Whereas the wild-type virus can reach high titers in culture, this attenuated version (MOPV-ExoN6b) reaches titers that are two logs lower [86], so it may not be vigorous enough for scaled production. In addition, it is possible that the altered exonuclease would allow vaccine persistence in vivo*.*

No live-attenuated RNA virus vaccines are recommended for administration to pregnant women because all replicating RNA viruses tested have been teratogenic for live births from an infected pregnant host. This is likely because viral replication requires host molecules that are also needed for fetal development [92]. Amongst the Lassa vaccines listed above, the first three are live-attenuated RNA viruses, whereas the last two, MVA and DNA vaccines, are not able to replicate in mammals, so are likely to be safe for pregnant human subjects. 

All five listed vaccines can be altered by reverse genetics. A recombinant ML29 (rML29) has recently been rescued from cDNA clones. Using rML29 and tri-segmented technology, additional genes of interest (eGFP, Ebola GP, and *Plasmodium berghei* antigens) have been expressed in rML29 [93]. Thus, rML29 can be used as a potent vaccine platform for expressing arenaviral genes (e.g., LASV GP_Pinneo_ from distantly-related lineage I) and non-related antigens and immunomodulators. 

At this time, only two Lassa vaccine candidates (ML29 and VSV-Las_GP_) have demonstrated breadth of protection in guinea pigs; and only one, ML29, has shown the ability to protect against challenges from virus outside the lineage IV of Lassa_Josiah_. After a head-to-head comparison, the ML29 and VSV-Las_GP_ candidates should move forward in clinical trials targeting health care workers and other people on the front lines of an outbreak. The two vaccine candidates that do not replicate in mammals, MVA-LAS and LASV-DNA, and would consequently be more expensive to produce, should be reserved for clinical trials with children and pregnant women.

## 6. Improving the Humoral Immune Response to Lassa Vaccines

The humoral immune response appears late after infection and after experimental vaccination. HIV, LCMV, and Lassa viruses evade antibodies by mimicking self-glycoproteins and cloaking their foreign envelope glycoproteins with self-glycans [94,95,96,97]. The development of B cell responses to glycan-cloaked epitopes is a slow process that ultimately depends upon antigen density and the avidity of the antigens for the B cell receptors (BCR). Self-reactivity can be removed from antibodies by V(D)J recombination or hypermutation. For example, binding studies showed that only three mutations in a BCR could confer 50-fold lower binding to self-versus foreign antigens [98]. Helper T cells cooperate with anergic B cells only when BCR cross-linking by foreign antigen is greater than that induced by self-antigen [99]. The higher threshold to activate anergic B cells and recruit them to germinal centers can only be overcome by high antigen density or high affinity for the BCR. This means that low density GP on virions will fail to activate anergic B cells, especially if they have only moderate affinity for self-glycans. Consequently, the B cell arm of the antiviral immune response will only develop after exposure to high-density, high-affinity antigen. 

Despite an initial cloaking of B cell antigens during viral infection, survivors acquire memory responses that increase over time. A study of 45 people who survived avian influenza showed that the sickest individuals, presumably those with highest viral titers, were the slowest to develop cell-mediated and humoral responses, but then they also developed the most long-term protective antibody responses [100].

The effort to make “universal flu vaccines” illustrates some approaches that could be used in making broadly-protective antibodies for Lassa virus disease. Initially, influenza researchers tried to increase the vaccine breadth by choosing immunogens such as the influenza hemagglutinin (HA) stem that are most evolutionarily conserved. By choosing conserved antigens, one could vaccinate against the most stable portions of a pathogen and also achieve the greatest cross-protective immune responses. In one particular effort, a broadly-reactive vaccine was created by engineering a consensus of 2656 HA_H1N1_ protein sequences into one protein, CH1, that bore conserved B and T cell epitopes. A PR8-CH1 influenza virus elicited broadly-protective immunity against heterologous H1N1 viruses [101]. Researchers also considered using the neuraminidase (NA) since it is a less rapidly-evolving protein and thus a good contributor for vaccine antigens. Similarly, for arenaviruses such as LASV, the GP1 is highly variable, but a segment of GP2 and the NP are not very variable and therefore good antigens to include as immunogens. 

It was recognized in the 1990s that the three-dimensional structure of an antigen contributed to the B cell response [102]. The contribution of large repetitive structures and conformational antigens to broadening the antigen response is an important approach that has been pursued in the search for universal influenza vaccines. Particulation of an antigen (e.g., putting it on a nanoparticle) leads to significant enhancement of BCR cross-linking and B cell activation [103]. B cell activation also leads to more activated CD4+ T cell responses, and promotion of antigen cross-presentation for CD8+ T cell activation [104]. Particulate antigens tend to cause auto-immunity, they are more frequently taken up by phagocytosis, they are DC-tropic, they are more immunogenic than soluble antigens, and they activate the inflammasome [105]. In agreement with these observations about particulation, vaccines comprised of virus particles or virus-like particles would be predicted to be more immunogenic than DNA vaccines or unstructured assemblages of viral antigens. 

The production of VLP also exploits the particulation approach. The MVA-VLP vaccine results in highly structured antigens, in this case the Lassa Z (or matrix protein) in combination with the Lassa GP form VLP in vivo [87]. Stable particle formation for retroviruses also depends on two types of proteins: the envelope (Env) is thought to bear the most variable “type-specific antigens” while the abundant capsid protein “Gag” bears the more evolutionarily-stable “group-specific antigens” [106]. By adding Gag to a vaccine some investigators have been able to introduce evolutionarily-conserved regions that also contribute to a more stable ribonucleoprotein (RNP) structure [107].

The most conserved antigenic regions tend to include many non-linear (conformational) antigens that remain functionally stable during virus evolution. This finding is strengthened by the observation that roughly 60% of the neutralizing antibodies derived from convalescent Lassa patients relied on conformational epitopes [108]. A remarkable effort to characterize nAbs from convalescent human blood revealed that as antibodies matured over time, some antibodies were capable of neutralizing Lassa isolates from all four lineages, and some could neutralize LCMV as well as Lassa [108]. Unfortunately, it is well known that pseudotypes are more sensitive to nAbs than wild-type viruses [109,110], so this conclusion needs corroboration with LASV isolates. In these cases of broadly-neutralizing antibodies, breadth of neutralizing activity was dependent on conformational epitopes. It is a common theme that broadly-neutralizing anti-microbial antibodies bind to conformational epitopes, for example one of the broadest and most potent HIV neutralizing antibodies binds to the gp41–gp120 interface [111].

Leon et al., [112] showed that binding of large structured antigen-antibody complexes to Fc gamma receptor (FcγR) leads to stronger and more broadly cross-reactive B cell responses than antigens that elicit FcγR-independent antibodies. Antibodies that were hemaglutination (HA)-inhibiting tended to be FcγR-independent and antibodies that bound the conserved HA stalk structure tended to be FcγR-dependent. There was a large discrepancy between in vitro and in vivo assays for nAb efficacy: in vitro, HA stalk antibodies were 100–1000 fold weaker than HA-inhibiting antibodies, but in vivo passive transfer studies showed only a five-fold discrepancy between antibody efficacies. By testing a number of HA variants, this group found that the optimal nAb response requires, not only an interaction with FcγR, but also a second molecular bridge between pathogen and innate effector cells, serving perhaps to activate those cells. Antibodies against the influenza HA are more broadly reactive if they simultaneously engage both the FcγR and other pathogen-specific receptors on innate immune cells [112]. To translate this finding to enhancing the breadth of Lassa vaccines, those vaccines that engage both viral entry receptors and FcγR on innate immune cells should be the most broadly cross-protective immunogens. For example, a recent publication illustrates how a beta-propriolactone-inactivated Lassa-Rabies vector elicits lasting humoral responses against LASV and Rabies in mice and guinea pigs [113].

## 7. Improving the Cell-Mediated Immune Response to Lassa Infection

Memory T cells play a critical role in long-term protection against Lassa fever [74]. Nevertheless, the CD8+ T cell receptor (TCR) interaction with MHC-presented peptides is very constrained. 9mer epitopes in the context of MHC-class I must have a very tight and specific fit to the TCR to activate cytokine secretion or CTL activity. One strategy known as the “string of beads” approach was meant to increase vaccine breadth by including many of the primary epitopes in one expression vector. This was tried for AIDS and for arenavirus vaccines, but it resulted in a competition between similar epitopes so that the immune response became quickly focused but not broader [114]. A slightly different approach, stringing the non-dominant epitopes, succeeded in avoiding focus and developed a broader protective vaccine response [115]. Using adenoviral vectors expressing invariant chain-linked non-dominant LCMV GP antigen, the AR Thomsen and JP Christensen laboratories were able to show that efficient virus control may be obtained by targeting the intrinsically non-dominant GP antigen, and that this allows for a potent CD8 T cell response to be elicited by virus-encoded dominant NP antigen during the chronic phase of a high-dose infection. In contrast, when mice were initially vaccinated using the dominant NP antigen, the subsequent virus-elicited response remained focused on the major NP epitope. During the early period after virus challenge, it was possible to confirm that T cells primed by the Adeno-GP vaccine and boosted by the virus infection were able to protect against a broad array of challenge viruses [115]. 

Whereas class I peptides present to CD8 T cells and are constrained in size to anchor within the peptide groove of MHC class I molecules, class II peptides present to CD4 T cells and are much less constrained, varying from 11 to 30 amino acids in length [116]. An approach suggested by Dr. M. Patarroyo for synthetic-vaccine development is to determine immune protection-inducing protein structures (IMPIPS) by stringing together class II peptides. This involves defining the three-dimensional interactions which are essential in MHC_ClassII_–peptide–Tcell receptor (TCR) complex formation, a much more promiscuous coupling activity than the interactions of class I peptides and CD8 TCR. After finding an orientation for perfectly fitting into the TCR that induces an appropriate immune response, non-interfering, long-lasting, protective, multi-epitope peptides can be synthesized against many infections [117,118]. Even non-immunogenic epitopes can be converted into immunogens using the Patarroyo TCR-binding selections, thereby avoiding the over-reaction problems caused by pre-existing immunity against microorganisms. 

In AIDS vaccine research, there has also been an approach favoring class II epitopes. This is promising because it is well known that CD4+ TCR can be more frequently cross-restrictive than the CD8 TCR [116]. The ability to cross-restrict allowed AIDS Elite Controllers to have both avid TCR interactions with Gag293 epitope in the context of HLA-DR (class II) and to have broad cross-restriction between Gag and Env V2 epitopes. Thus, a broad cross-reactivity of CD4+ T cell responses, similar to that commonly seen in Elite Controllers, could be established by vaccination in ordinary individuals [119]. With respect to Lassa vaccines, it is likely that Lassa disease survivors bear some similarities to AIDS Elite Controllers in their acquired immunity, showing broadly cross-restricted CD4 responses. An understanding of the CD4 TCR alleles determining this response should make it possible to engineer autologous T cells that confer protection.

With high HLA heterogeneity among the West African population, the feasibility of epitope-based vaccines is limited. A safety issue was raised in murine experiments where epitope-based vaccines given to individuals who had previously been exposed to the pathogen can strongly re-activate the pre-existing CD8+ T clones and induce TNF-dependent immunopathology [120], but then this may be a murine problem and not a serious problem for human subjects.

## 8. Future Directions Combining Immunological and Drug-Therapy Approaches

When health care professionals are getting ready to risk their lives during an epidemic, a strong, fast-acting and broadly-protective vaccine should be available. The poor conditions and shear volume of needy patients makes a dangerous situation for first-responders and they deserve to be armed with protection. In addition to vaccines, broadly-protective antiviral therapies should be available. Both the ML29 and VSV-LAS vaccines have shown antiviral efficacy when used within two days of Lassa infection [16,81], however their action within this brief window of time is lineage-specific rather than broadly protective. It is telling that a repatriated health care worker who ultimately survived was given intravenous fluids and two broadly acting antivirals (ribavirin pn days 6–15 of illness and favipiravir days 8–12 of illness) [74]. Nevertheless, it is still unclear whether the optimum care requires anything more than intravenous fluids and good hospital practices. 

Several studies have yielded broadly cross-protective antivirals that could be used against Lassa infection. High-throughput screens for small molecule inhibitors of LASV revealed an inhibitor of arenavirus polymerases (favipiravir) that was recently used on a LASV-infected health care worker along with intravenous fluids and ribavirin [74]. This treatment is so broadly cross-protective it has even been used on Ebola cases [121]. Ribavirin also has broad reactivity against many RNA viruses and at its lowest concentration works by blocking the capped-mRNA-promoting function of eIF4E [122]. Unfortunately, ribavirin resistance is problematic; thus, it is best used in combination with other antivirals [123]. A recent study of the interactome of LCMV gene products with host cell molecules revealed several host-cell molecules essential to the life cycle of viruses including Junin, LASV and Ebola [124,125]. Mining such data will yield a steady pipeline of antiviral approaches that could protect simultaneously against several viruses. The standard of care for patients may eventually include both rapid-acting vaccines and broadly-acting antiviral drugs. 

In summary, there is an urgent need for head-to-head comparisons of the current popular vaccine candidates because their relative stabilities, production capacities, and protective breadth in primates remain unknown. Having established such a comparison, two types of vaccines should be developed: a cheaply-produced vaccine (e.g., ML29 or VSV-LAS) for emergency use in endemic areas, and a more expensive vaccine (e.g. the MVA or DNA vaccines) for vulnerable populations such as pregnant women. These two types of vaccines can be further improved by increasing immunity to conformational antigens or by favoring epitopes known to bind tightly to HLA and cross-restrict with conformational antigens. After following this path to improving the current popular Lassa vaccine candidates, treatments combining vaccines and therapeutic drugs should be optimized as well.

## Figures and Tables

**Table 1:** Comparison of select Lassa vaccine candidates.

Vaccine	Breadth of Cross-Protection ^a^	Safety for Pregnant Women and Fetus ^b^	Sterilizing Immunity ^c^	Production Costs ^d^	References
ML29 ^e^	High	Low	Yes	Low	[16]
MOPVAC_LasGP_^f^	ND ^g^	Low	Yes	Med	[86]
VSV-LAS_GP_^h^	Med	Low	No	Med	[81]
MVA-LAS_GP+Z_^i^	ND	High	ND	Med	[87]
LASV_GPC_ DNA ^j^	ND	High	Yes	High	[88]

^a^ Refers to protection from distantly-related virus isolates. ^b^ This is a guess based on the propensity of similar viruses to cause fetal malformations or miscarriage. ^c^ Sterilizing immunity means that, after immunizing with an effective dose, there is no trace of the vaccine a week after immunization, neither in tissue nor in excreta. ^d^ Production costs are extrapolated from reported doses and levels of virus (or RNA) production in cell culture. ^e^ Mopeia/Lassa reassortant 29 (ML29) has the L RNA of Mopeia and the S RNA of Lassa_Josiah_. It was selected from a library of MOPV/LASV reassortants for small-plaque phenotype, attenuation in mice, genotype from MOPV L RNA and LASV S RNA, genetic stability, and efficient replication in Vero cell cultures (~10^8^ plaque forming units (pfu)/mL) [14]. The Russian laboratories of Fort, LLC have produced a variety of Mopeia/Lassa reassortants to improve upon the vaccine efficacy and patent protections of the initial isolates (Moshkoff D. and Nasidi A. in preparation). ^f^ MOPVAC or Mopeia-ExoNb6 (MOPV-ExoNb6) is a recombinant virus expressing the Mopeia genome, six mutations in the MOPV-NP exonuclease, and the LASV GP in place of the MOPV GP [86]. ^g^ ND means not determined. ^h^ VSV-LAS_GP_ refers to vaccines using the vesicular stomatitis virus (VSV) platform and expressing the Lassa GP. Both current versions have reduced neurovirulence: the Feldmann/Merck version has been attenuated by replacing the VSV G with the Lassa GP [89], and the Rose/Profectus version has been attenuated by altering the natural VSV gene order [90]. Both have reduced growth capacity compared to VSV, and, from our experience with other VSV pseudotypes, it is likely that they fail to reach titers above 10^7^ plaque-forming units (pfu)/mL. ^i^ The GeoVax-made vaccine replicates well in avian cells but does not replicate in mammals. In mammals, it expresses LASV GP and Z genes, forming virus-like-particles (VLP) in vivo. The Modified Vaccinia Ankara (MVA) vector was developed by B. Moss at NIH and has been used in thousands of human beings in the form of an AIDS vaccine [91]. VLP can be powerful and broadly-protective immunogens. ^j^ LASV_GPC_ DNA vaccine.
